# The Role of *Candida albicans* Transcription Factor *RLM1* in Response to Carbon Adaptation

**DOI:** 10.3389/fmicb.2018.01127

**Published:** 2018-05-29

**Authors:** João Oliveira-Pacheco, Rosana Alves, Augusto Costa-Barbosa, Bruno Cerqueira-Rodrigues, Patricia Pereira-Silva, Sandra Paiva, Sónia Silva, Mariana Henriques, Célia Pais, Paula Sampaio

**Affiliations:** ^1^Department of Biology, CBMA, School of Sciences, University of Minho, Braga, Portugal; ^2^UCD School of Biomolecular and Biomedical Science, University College Dublin, Dublin, Ireland; ^3^ICVS, School of Health Sciences, University of Minho, Braga, Portugal; ^4^CEB, School of Engineering, University of Minho, Braga, Portugal

**Keywords:** *C. albicans*, candidiasis, carbon adaptation, lactate, alternative carbon sources, *RLM1*, cell wall remodeling

## Abstract

*Candida albicans* is the main causative agent of candidiasis and one of the most frequent causes of nosocomial infections worldwide. In order to establish an infection, this pathogen supports effective stress responses to counter host defenses and adapts to changes in the availability of important nutrients, such as alternative carbon sources. These stress responses have clear implications on the composition and structure of *Candida* cell wall. Therefore, we studied the impact of lactate, a physiologically relevant carbon source, on the activity of *C. albicans RLM1* transcriptional factor. *RLM1* is involved in the cell wall integrity pathway and plays an important role in regulating the flow of carbohydrates into cell wall biosynthesis pathways. The role of *C. albicans RLM1* in response to lactate adaptation was assessed in respect to several virulence factors, such as the ability to grow under cell wall damaging agents, filament, adhere or form biofilm, as well as to immune recognition. The data showed that growth of *C. albicans* cells in the presence of lactate induces the secretion of tartaric acid, which has the potential to modulate the TCA cycle on both the yeast and the host cells. In addition, we found that adaptation of *C. albicans* cells to lactate reduces their internalization by immune cells and consequent % of killing, which could be correlated with a lower exposure of the cell wall β-glucans. In addition, absence of *RLM1* has a minor impact on internalization, compared with the wild-type and complemented strains, but it reduces the higher efficiency of lactate grown cells at damaging phagocytic cells and induces a high amount of IL-10, rendering these cells more tolerable to the immune system. The data suggests that *RLM1* mediates cell wall remodeling during carbon adaptation, impacting their interaction with immune cells.

## Introduction

*Candida albicans* is an opportunistic pathogenic fungus responsible for a wide spectrum of infections in immunocompromised individuals, ranging from superficial mycosis to systemic and disseminated candidiasis (Pfaller and Diekema, 2007; Brown et al., 2012). These infections are estimated to cause 400,000 deaths each year, remaining by far the most common of all invasive fungal infections (Brown et al., 2012; Campion et al., 2015).

This pathogen thrives within distinct niches in the human host, including the skin, the oral cavity, the gut, and the genitourinary tracts (Southern et al., 2008). These niches differ considerably in terms of nutrients, pH, and local microbiota and, in order to survive and proliferate, *C. albicans* must adapt to the changing host environment. This extraordinary flexibility to adapt to the different environmental conditions activates the expression of several virulence factors and affects the resistance of this pathogen to multiple stresses (Brown et al., 2014; Hall, 2017; Miramón and Lorenz, 2017).

Like most microorganisms, *C. albicans* possesses a dynamic cell wall that responds efficiently to host-imposed stresses, including changes in carbon sources (Ene et al., 2012a, 2013; Ballou et al., 2016) or exposure to antifungal drugs (Wheeler et al., 2008). This protection is conferred by a carbohydrate-based matrix containing chitin, β-glucans, and mannoproteins, each of which has an important role in innate immune recognition (Netea et al., 2008). For instance, the recognition of β-glucans by the receptor dectin-1, which is present at the cell surface of immune cells, promotes phagocytosis and killing by macrophages and neutrophils (Brown and Gordon, 2001; Hardison and Brown, 2012). Consequently, any change in the structure of cell wall will therefore impact innate immune recognition and virulence (Hall, 2017).

Much of what is known about the fungal cell wall integrity (CWI) results from studying the yeast model *Saccharomyces cerevisiae*, where the CWI mitogen-activated protein kinase pathway (also known as the PKC pathway) is the main system responsible to repair the cell wall and maintain the cell integrity (Lipke and Ovalle, 1998; Levin, 2011). The targets of the CWI pathway activation are the Swi4-Swi6 cell cycle box-binding factor (SBF) (Baetz et al., 2001) and the major effector the MADS-box transcription factor *RLM1* (Dodou and Treisman, 1997; Watanabe et al., 1997; Jung et al., 2002). Although this pathway is conserved in *C. albicans*, the role of *RLM1* as the main transcriptional regulator of the cell wall stress responses is not conserved in this pathogenic species and other additional transcription factors, such as Cas5, have been proposed as key regulators in this pathway (Bruno et al., 2006; Rauceo et al., 2008; Blankenship et al., 2010; Xie et al., 2017). Even so, *C. albicans RLM1* gene has been shown to be required for cell wall integrity, at least under Caspofungin, Calcofluor White, and Congo Red stresses (Bruno et al., 2006; Delgado-Silva et al., 2014). This gene has also an increased genetic variability that has been associated with strain susceptibility to different stress conditions, with some genetic variations enhancing resistance (Sampaio et al., 2009). Additionally, the absence of *RLM1* alters the cell wall content, specifically the chitin and the mannan layers, increasing cell adhesion *in vitro* and reducing virulence *in vivo* (Delgado-Silva et al., 2014). Some findings also suggested that this gene participates in the cell wall biogenesis, particularly in regulating the flow of carbohydrates into cell wall biosynthesis pathways (Delgado-Silva et al., 2014).

Here, we explore the involvement of *C. albicans RLM1* on cell wall biogenesis and virulence during carbon source adaptation. To approach this, *C. albicans* cells were grown in the presence of lactate, a particularly abundant metabolite in several host niches (Owen and Katz, 1999; Barelle et al., 2006). Exposure to the alternative carbon source lactate is particularly relevant as it has been shown to affect the cell wall architecture of *C. albicans* (Ene et al., 2012a, 2013; Ballou et al., 2016). In order to understand whether *RLM1* is involved in this process, two *RLM1* mutant strains adapted to lactate were characterized with respect to several virulence factors, such as the ability to grow under cell wall damaging agents, filament, adhere, or form biofilm. The involvement of *RLM1* in host-pathogen interaction was also assessed, providing new insights into the role of *C. albicans RLM1* in cell wall regulatory responses and pathogenicity.

## Materials and Methods

### Strains and Growth Conditions

Five *C. albicans* strains were used during this study: the wild-type SC5314 strain (Gillum et al., 1984), two *RLM1* mutant strains (SCRLM1M4A and SCRLM1M4B), and two *RLM1* complemented strains (SCRLM1K2A and SCRLM1K2B) (Delgado-Silva et al., 2014). All strains were stored as frozen stocks with 30% (v/v) glycerol and routinely cultured on YPD agar plates (1% yeast extract, 2% peptone, 2% dextrose, and 2% agar) stored at room temperature. For all experiments, a pre-inoculum was prepared by collecting one colony from the YPD plate and allowing the cells to adapt to minimal medium containing either 2% glucose or 2% lactate, and 0.67% yeast nitrogen base (YNB) without amino acids (pH 5.2–5.6) at 30°C, overnight (for glucose) or during 24 h (for lactate). The inoculum was then prepared with adapted cells into new medium, diluting the cells to an optical density (OD_600nm_) of 0.1, and allowing the cells to grow.

### High-Performance Liquid Chromatography (HPLC)

*Candida albicans* cells were grown in YNB 2% glucose or YNB 2% lactate (30°C, 200 rpm) as previously described and, at different time points, 1 mL of the cell cultures were harvested and centrifuged (5 min, 5000 *g*). The supernatants were then prepared and analyzed for the detection of different organic acids, glucose, glycerol, and ethanol by HPLC. Culture supernatant samples were treated with 10% trichloroacetic acid to remove protein contaminants, centrifuged for 15 min at 14,000 rpm, and then filtered through a 0.22-μm filter before analysis. HPLC analysis was performed in a Rezex 8 μm ROA-organic acid H+ (8%) high-performance liquid chromatography column (Phenomenex) with an Elite LaChrom (VWR Hitachi) chromatography system, according to Collins et al. (2014). A total of 2.5 mM H_2_SO_4_ was used for the mobile phase, the column was maintained at 60°C, and detection was by refractive index measurement with an Elite LaChrom L-2490 RI detector (VWR Hitachi) at 40°C. Samples from at least five independent replicates were analyzed.

### Susceptibility Assays

Fungal cells were incubated overnight in YNB 2% glucose or YNB 2% lactate (30°C, 200 rpm), diluted to OD_600nm_ = 0.05–0.1 and left to grow until OD ∼1 with fresh medium. Drop tests were performed by spotting 5 μL of the serially diluted cell suspension onto YNB 2% glucose or 2% lactate agar plates supplemented with the following compounds: 200 μg.mL^-1^ Calcofluor White, 100 μg.mL^-1^ Congo Red, 90 ng.mL^-1^ Caspofungin, 10 mM Caffeine and 0.035% (w/v) SDS. Plates were incubated for 48 h at 30°C before observation. A minimum of three independent replicates was performed.

### Filamentation Tests

All strains were grown overnight in YNB 2% glucose or YNB 2% lactate (30°C, 200 rpm), diluted to OD_600 nm_ = 0.05–0.1 and left to grow until OD ∼1 with fresh medium. Cell were then spin down, rinsed two times with PBS, diluted to the same volume with Dulbecco’s modified Eagle’s medium (DMEM) and incubated at 37°C and 5% CO_2_. In order to observe the filamentation, yeast cells were stained with Calcofluor White and monitored by fluorescence microscopy (Leica, DM5000B). Hyphal length was then measured with ImageJ 1.51s (NIH, United States). A minimum of 50 cells from each condition was measured in images taken from three independent replicates.

### Adhesion and Biofilm Formation Assays

To determine the impact of different carbon sources on adhesion and biofilm formation ability, 24-well microplates (Orange Scientific, Braine-l’Alleud, Belgium) were filled with *C. albicans* cell suspensions (1 mL containing 1 × 10^6^ cells) grown on each carbon source until reaching stationary growth phase (20 h for glucose-grown cells and 40 h for lactate-grown cells) as described above, and incubated at 37°C, 120 rpm. Adhesion and biofilm formation were assessed through quantification of total biomass by crystal violet (CV) staining (Stepanovic et al., 2000). The measurements were performed after 2 h of incubation for adhesion ability and the biofilm formation was assessed after 24 and 48 h. At 24 h, 500 μL of cultured medium was removed and replaced by fresh medium. After the defined times of incubation, the medium was aspirated and non-adherent cells removed by washing the wells with sterile ultra-pure water. For total biomass quantification, cells were fixed with 1 mL of methanol during 15 min. After that, the methanol was removed, the plates were allowed to dry at room temperature and 1 mL of CV (1% v/v) was added to each well. After 5 min, the wells were gently washed with sterile, ultra-pure water and 1 ml of acetic acid (33% v/v) was added to release and dissolve the stain. The absorbance of the obtained solution was read in triplicate in a microtiter plate reader (SpectraMax Plus) at 570 nm. Results were presented as absorbance/area of the wells (abs.cm^-2^) from three independent replicates.

### Scanning Electron Microscopy (SEM)

The biofilm structure was observed by SEM. For that, biofilms were formed in 24-well polystyrene microtiter plates (Orange Scientific, Braine-l’Alleud, Belgium) with 1 mL of 1 × 10^5^ cells suspensions, as described previously. After 48 h of incubation the formed biofilms were washed with PBS, dehydrated with alcohol (using 70% ethanol for 10 min, 90% ethanol for 10 min, and 100% ethanol for 20 min) and air-dried. Prior to observation, the base of the wells was mounted onto aluminum stubs, sputter coated with gold, and observed with an Ultra-high resolution Field Emission Gun Scanning Electron Microscopy (FEG-SEM; Nova NanoSem 200, FEI Company, United States).

### Quantification of β-Glucan Exposure

Quantification of β-glucans was performed as described previously (Ballou et al., 2016), with some modifications. Briefly, *C. albicans* cells were grown in YNB 2% glucose or YNB 2% lactate and collected at stationary phase, as described above. A total of 2.5 × 10^6^ cells were counted with a hemocytometer, and washed with cold FACS buffer (1 × PBS, 1% FBS, 0.5 mM EDTA). Cells were then resuspended in 100 μl FACS buffer + 5 ng.μL^-1^ anti-β-1,3-glucans (BioSupplies) and incubated in the dark on ice for 1 h. Cells were washed (5000 rpm, 3 min) twice in FACS buffer, resuspended in 100 μL FACS buffer plus 1:200 anti-mouse IgG conjugated to Alexafluor 647 (Invitrogen) and incubated in the dark on ice for 1 h. Cells were washed as above, fixed in 4% formaldehyde, diluted in FACS buffer, washed again, and analyzed by flow cytometry (BD LSR II, Becton Dickinson) using FACSDiva software. For each experiment, at least 20,000 events were acquired for each sample. As a control, aliquots from all cells to be analyzed were pooled, diluted to 2.5 × 10^6^ cells and treated as above except that no anti-β-1,3-glucans was added. Median fluorescence intensities (MFIs) were determined using FlowJo software (Tree Star, v 10.2) and reported for each sample. Plots are representative of two independent assays.

### Phagocytosis Assays

All experiments were conducted with the approval of the Ethical Committee Board of the Portuguese Veterinary Directorate and they all adhered to local and institutional policy requirements. Single-cell suspensions of bone marrow cells were prepared by aseptically removing femurs from C57BL/6J wild-type mice. Bones were cut on both ends and marrow was flushed with ice-cold supplemented RPMI 1640 (10% heat-inactivated FBS, 10 mM HEPES, 1 mM NaPyruvate, 2 mM L-glutamine, 50 mg.ml^-1^ streptomycin, and 50 U/mL penicillin, all from Merck). Bone marrow cells were then resuspended in RPMI 1640 supplemented with M-CSF (20 ng.mL^-1^ Peprotech) and seeded in a 24-well plate at 5 × 10^5^ cells/well. Cells were incubated for 7 days at 37°C with 5% CO_2_. After 4 days of incubation, 1 mL of fresh RPMI 1640 supplemented with M-CSF was added. For fluorescence microscopy assays, macrophages were incubated in the presence of sterile glass coverslips (diameter 13 mm). On the day of co-culture, macrophages were washed with sterile PBS and fresh medium was added. *C. albicans* grown in YNB 2% glucose or YNB 2% lactate until stationary phase, as described before, were fixed with formol/ethanol (1:9) for 10 min, washed five times with sterile PBS, and incubated for 10 min with Sytox Green at room temperature in the dark. Yeast cells were then washed with sterile PBS to remove unbound dye and brought to the desired cell density in RPMI 1640. Macrophages were incubated with labeled yeast suspensions at a multiplicity of infection (MOI) of 5Y:1M ratio, for 30 min at 37 °C and 5% CO_2_. After incubation plates were kept on ice to stop phagocytosis, and wells rinsed twice with PBS to remove unbound yeasts. Macrophages and associated yeasts were then incubated with Propidium Iodide (PI) at a final concentration of 6 μg.mL^-1^, for 5 min at room temperature (Carneiro et al., 2014). Cells were analyzed by flow cytometry (BD LSR II, Becton Dickinson) and by fluorescence microscopy (Leica DM5000B). Cytometry data was analyzed using FlowJo software (Tree Star, v 10.2) and fluorescence microscopy images using ImageJ cell counter software. All experiments were done in duplicate and results were obtained from three independent experimental assays.

### Host Viability Assays

The yeast killing assay was performed as previously described by (McKenzie et al., 2010). Macrophages and yeast cells adapted to each carbon source, were cultured in 96-well tissue culture plate (SpectraMaxPlus), and incubated for 1 h at MOI 5Y:1M ratio. After incubation, the 96-well tissue culture plate was centrifuged for 2 min at 750 *g* and 80 μL of supernatant was transferred to a new 96-well microplate and stored at -80°C for further analysis. The final volume was restored by adding 80 μL of 10% saponin followed by gently up and down pipetting in order to lyse macrophages and release the adhered cells. Wells with *Candida* alone and incubated in the same conditions represented 100% viability. Serial 10-fold dilutions were then plated on YPD agar plates and incubated at 30°C for 24 h. Lactate dehydrogenase (LDH) activity was measured in the supernatant of the yeast killing assay. Each reaction contained 40 μL of extracellular LDH, 250 μl of NADH (0.28 mM), and 10 μL of pyruvate (0.32 mM). Both NADH and pyruvate solutions were prepared in 0.05 M phosphate buffer pH 7.4. NADH conversion to NAD^+^ was spectrophotometrically evaluated in a microplate reader (Molecular Devices, SPECTRAmax Plus 384) at 340 nm, every 10 s for 3 min, at 30°C (Korzeniewski and Callewaert, 1983). MTT assay was used to evaluate metabolic activity of 10^5^ cells/mL macrophages (cell line RAW 264.7) with tartaric acid (0.075 g/L). Briefly, after tartaric acid incubation, for 24 and 72 h, the supernatants were removed and cells were incubated with MTT solution (final concentration 0.45 mg/mL) for 2 h at 37°C and 5% CO_2_. Then, the supernatant was discarded, the formazan crystals resuspended in DMSO/Ethanol 1:1 (v/v) and final absorbance measured at 570 nm in the SPECTRAmax Plus 384 microplate reader. TNF-α and IL-10 levels were quantified using commercially available sandwich ELISA kits (Quantikine, R&D Systems, Abingdon, United Kingdom and KMC0102, Biosource, Camarillo, CA, United States, respectively) according manufacturer’s instructions. All experiments were carried out in triplicate.

### Statistical Analyses

Statistical analyses were performed using Graph Pad Prism (v. 7) and significance was determined using two-way ANOVA with Tukey’s multiple comparison test. All tests were performed with a confidence level of 95%.

## Results

### Characterization of *C. albicans* Growth and Metabolism on Lactate

We have proposed that the major role of *C. albicans RLM1* is in the biogenesis of the cell wall, particularly in regulating the flow of carbohydrates into cell wall biosynthesis pathways (Delgado-Silva et al., 2014). However, these conclusions were based in experiments using *C. albicans* cells grown on media containing 2% glucose, leaving the effects of other host carbon sources, such as lactate, largely unexplored. Here, we have used a set of *RLM1* null mutants previously constructed from the prototrophic wild-type model strain SC5314 (Delgado-Silva et al., 2014) to evaluate phenotype and impact on immune recognition when cells are exposed to different carbon sources: glucose or lactate.

**FIGURE 1:**
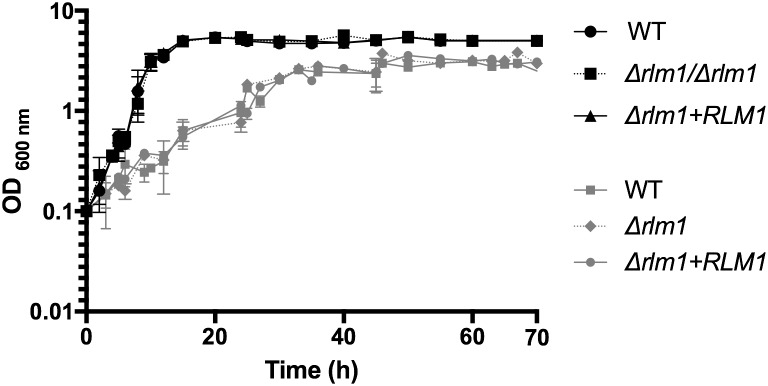
Growth of *C. albicans RLM1* wild-type, mutant, and complemented strains. *C. albicans* wild-type (WT), homozygous mutants (Δ*rlm1*/Δ*rlm1*), and complemented (Δ*rlm1+RLM1*) strains were grown in minimal YNB medium containing 2% of glucose (black lines) or lactate (gray lines), as the sole carbon source. Growth was monitored by optical density. Results presented are mean values and standard deviation (*n* ≥ 5).

Slow growth of the mutant strains may lead to differences in phenotypes, thus wild-type, mutant, and complemented *C. albicans* cells were grown on minimal medium in which the sole carbon sources were glucose or lactate (**Figure 1**). As we reported previously (Delgado-Silva et al., 2014), the growth rate of the different strains was unaffected in minimal medium with glucose (**Figure 1**). The doubling time of the mutant in glucose was 4.9 h, with no significant difference to the wild-type strain (4.5 h) or complemented strain (4.5 h). In contrast, growth was significantly slower for all strains, when lactate was the sole carbon source (**Figure 1**). The doubling time of the mutant in lactic acid was 17.7 h, with no significant difference to the wild-type strain (17.3 h) or complemented strain (16.5 h). Therefore, we can conclude that loss of *RLM1* did not affect growth rate on any carbon source.

**FIGURE 2:**
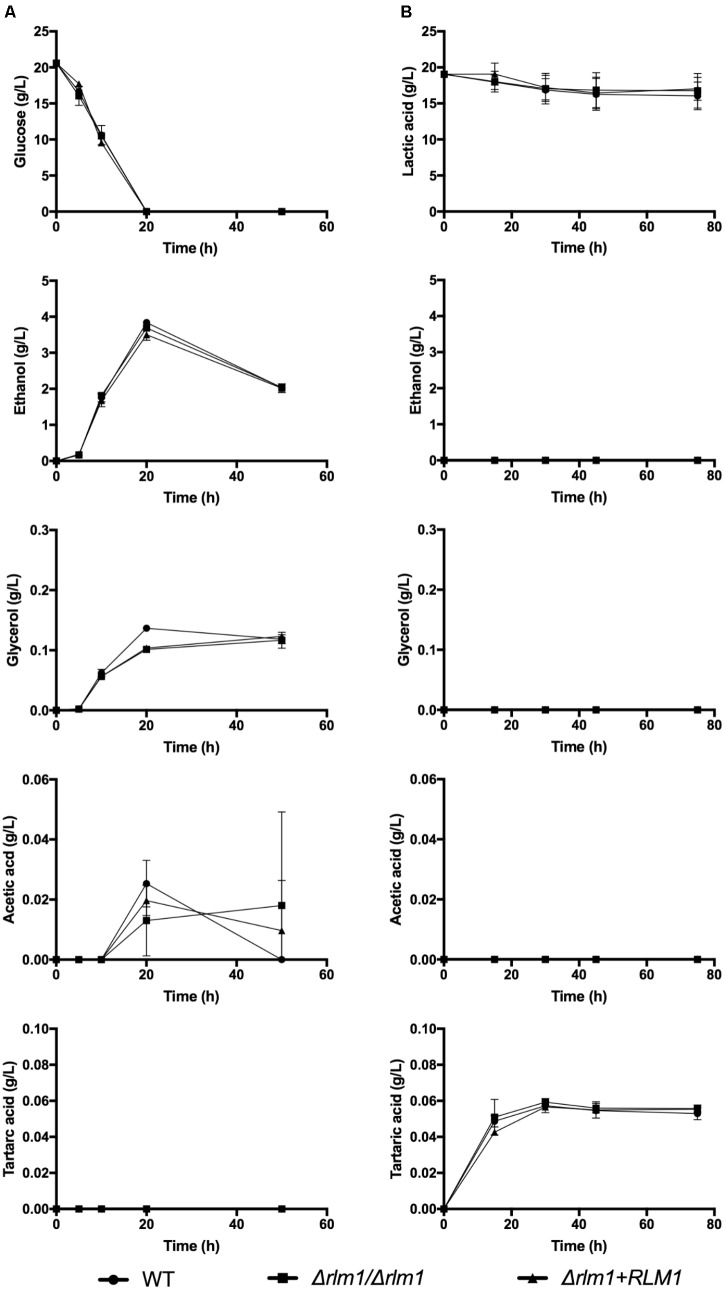
Identification of *C. albicans* metabolites during growth on different carbon sources by HPLC. *C. albicans* wild-type (WT), homozygous mutants (Δ*rlm1*/Δ*rlm1*), and complemented (Δ*rlm1+RLM1*) strains were grown in YNB 2% glucose **(A)** or lactate **(B)**. At different time-points glucose and lactic acid consumption and the variations in glycerol, ethanol, acetic acid and tartaric acid levels were monitored by HPLC. Results represent mean + SD (*n* ≥ 5).

We have also evaluated the production of several metabolites by the different strains during growth either in glucose or in lactate (**Figure 2**). After 20 h of growth in the presence of glucose, this carbon source was totally consumed and, as expected, all strains produced mainly glycerol, ethanol, and acetic acid (**Figure 2**). In contrast, during growth in lactate, the consumption of this carbon source was slower, stabilized after 45 h of growth and less than 20% of the initial amount was consumed (**Figure 2**). This consumption was significant after 45 h (*P* < 0.05) for the WT and complemented strains but not for the mutant (*P* = 0.2056). During this time, no ethanol, acetic acid, nor glycerol was detected (**Figure 2**), since this is a non-fermentative carbon source. Curiously, the production of a small amount of tartaric acid was observed only with lactate-grown cells (**Figure 2**). However, we can conclude that loss of *RLM1* did not significantly affect the metabolic usage of each carbon source.

### *C. albicans RLM1* Hypersensitivity to Congo Red Is Rescued by Growth on Lactate

**FIGURE 3:**
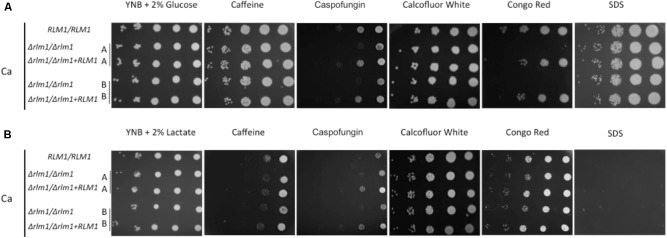
Sensitivity of *C. albicans* wild-type (WT), mutant (*Δrlm1/Δrlm1*), and complemented (rlm1Δ +*RLM1*) strains to several agents that affect cell integrity. **(A)** Serial 10-fold dilutions of overnight cultures were spotted on YNB 2% glucose or **(B)** YNB 2% lactate plates with 10 mM Caffeine, 90 ng.ml^-1^ Caspofungin, 200 μg.ml^-1^ Calcofluor White, 100 μg.ml^-1^ Congo Red, and 0.035% SDS for 2 days at 30°C. Images are representative of three independent experiments.

In order to evaluate the impact of an alternative carbon source on the role of *C. albicans RLM1*, we determined the sensitivity of the wild-type, Δ*rlm1*/Δ*rlm1* mutant and complemented strains to a range of cell wall-perturbing agents in the presence of glucose or lactate. In glucose medium, strains were able to grow well in the presence of all the compounds except in caspofungin, with the homozygous mutants being more sensitive. The mutant strains presented hypersensitivity to Congo Red when compared with complemented and parental strains (**Figure 3A**), as previously reported (Bruno et al., 2006; Delgado-Silva et al., 2014). In contrast, in the presence of lactate, all strains were hypersensitive to SDS, Caffeine, and Caspofungin (**Figure 3B**). In the absence of a functional *RLM1*, *C. albicans* cells grown in the presence of lactate showed more sensitivity to Caspofungin, when compared to their glucose counterparts. However, the hypersensitivity to Congo Red observed with these cells grown in glucose diminished greatly. This result indicates that when grown in lactate cells are more sensitive to cell wall stressing agents but, curiously, the absence of a functional *RLM1* rescues the hypersensitivity to CR observed in glucose grown cells (**Figure 3B**).

### The Transcription Factor *RLM1* Is Important for *C. albicans* Filamentation and Biofilm Formation

**FIGURE 4:**
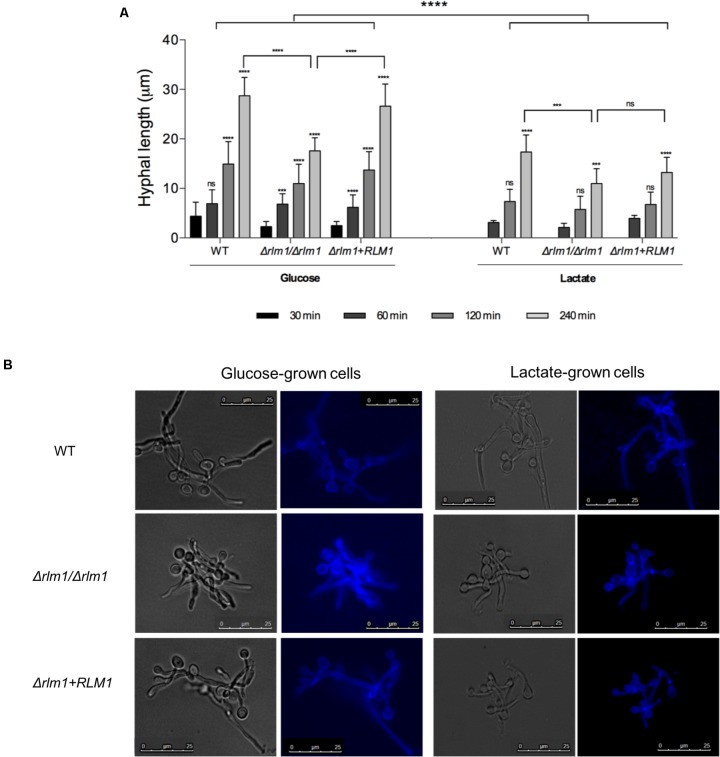
Filamentation of *C. albicans* wild-type (WT), mutant (*Δrlm1/Δrlm1*), and complemented strains (*Δrlm1*+*RLM1*). **(A)** Hyphal length of glucose and lactate-grown cells measured after 30, 60, 120, and 240 min of incubation in DMEM medium supplemented with 10% FBS at 37°C. Results represent mean + SD of three independent replicates. ^∗^*P* < 0.05, ^∗∗^*P* < 0.01, ^∗∗∗^*P* < 0.001, and ^∗∗∗∗^*P* < 0.0001 indicate statistically significant results; ns indicates not significant. For each strain and condition, *P*-values were always calculated in comparison with the previous time point. **(B)** Morphology of glucose and lactate-grown cells after 240 min of incubation in DMEM. *Candida* cells were stained with Calcofluor White and monitored by fluorescence microscopy. Images are representative of three independent experiments.

**FIGURE 5:**
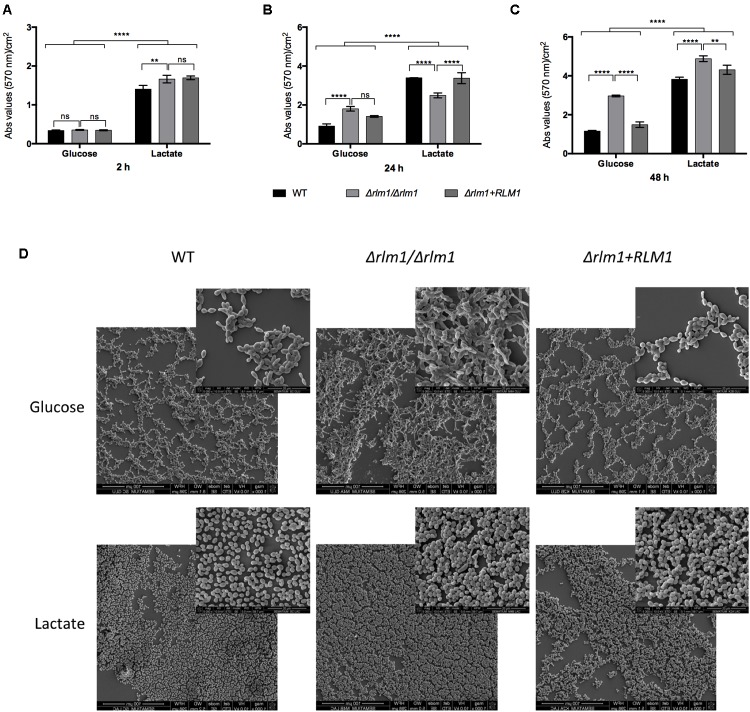
*In vitro* adhesion and biofilm formation of *C. albicans* wild-type (WT), mutant (*Δrlm1/Δrlm1*), and complemented strains (*Δrlm1*+*RLM1*). **(A)** WT, *Δrlm1/Δrlm1*, and *Δrlm1+RLM1* cells were allowed to adhere 2 h and **(B)** to form biofilm during 24 h and **(C)** 48 h in a polystyrene surface. Results represent mean values and standard deviation of three independent replicates (*n* = 3 per group per experiment). ^∗^*P* < 0.05, ^∗∗^*P* < 0.01, ^∗∗∗^*P* < 0.001, and ^∗∗∗∗^*P* < 0.0001 indicate statistically significant results; ns indicates not significant. **(D)** Representative SEM images of cells after 48 h of incubation.

Filamentation and biofilm formation represent two of the major virulence factors contributing to *Candida* pathogenesis. A previous work using Δ*rlm1*/Δ*rlm1* mutant strains grown on glucose-containing media has shown a higher upregulation of proteins involved in adhesion and biofilm formation (Delgado-Silva et al., 2014). Additionally, some studies have demonstrated that lactate-grown cells display higher ability to adhere and form biofilm when compared to glucose-grown cells (Ene et al., 2012b; Alves et al., 2017). Based on these studies, Δ*rlm1*/Δ*rlm1* mutant strains were tested regarding their ability to filament (**Figure 4**), to adhere to a polystyrene surface (**Figure 5A**), and to form biofilm after 24 h (**Figure 5B**) and 48 h (**Figure 5C**) of incubation, in the presence of lactate.

For filamentation analysis, glucose- and lactate-grown cells were incubated in induced media for 30, 60, 120, and 240 min, stained with Calcofluor White and monitored by fluorescence microscopy. Independently of the condition, all strains were able to filament and hyphae length increased with time. In glucose-grown cells, filaments were visible right after 30 min of incubation, while in lactate-grown cells only after 60 min (**Figure 4**). Moreover, lactate-grown cells presented shorter hyphae than glucose-grown cells. The lack of a functional *RLM1* affected filamentation of cells adapted to both carbon sources, and although the differences in hyphae length were visible in early time points, only after 240 min of incubation the differences were significant (*P* < 0.001). This difference was more pronounced in cells grown on glucose (**Figure 4**).

Then, all strains were evaluated regarding their ability to adhere to a polystyrene surface. After 2 h of incubation, lactate-grown cells showed a higher ability to adhere when compared with the glucose-grown cells (*P* < 0.0001), as previously reported (Ene et al., 2012b), and no differences were seen regarding the *Δrlm1/Δrlm1* mutant strain (**Figure 5A**). Considering biofilm formation, cells grown in lactate were able to form more biofilm than cells grown in glucose. As expected, Δ*rlm1*/Δ*rlm1* mutant formed more biofilm than the wild-type in presence of glucose at 24 and 48 h of incubation (**Figures 5B,C**). However, in the presence of lactate, at 24 h of incubation the mutant presented lower biofilm formation but at 48 h the amount of biofilm formed was higher than the WT and complemented strains (**Figures 5B,C**). SEM analyses confirmed the differences in biofilm formation after 48 h of incubation (**Figure 5D**). These results showed that lactate-grown cells presented higher biomass than their glucose counterparts and the mutant presented higher biofilm formation in both conditions, after biofilm maturation.

Overall, these results indicated that *RLM1* is important for filamentation, adhesion, and biofilm formation and that these phenotypes were similar for cells adapted to glucose or to lactate, suggesting that they are independent of the carbon source.

### *C. albicans RLM1* Does Not Affect Immune Recognition but Is Important for Immune Activation

**FIGURE 6:**
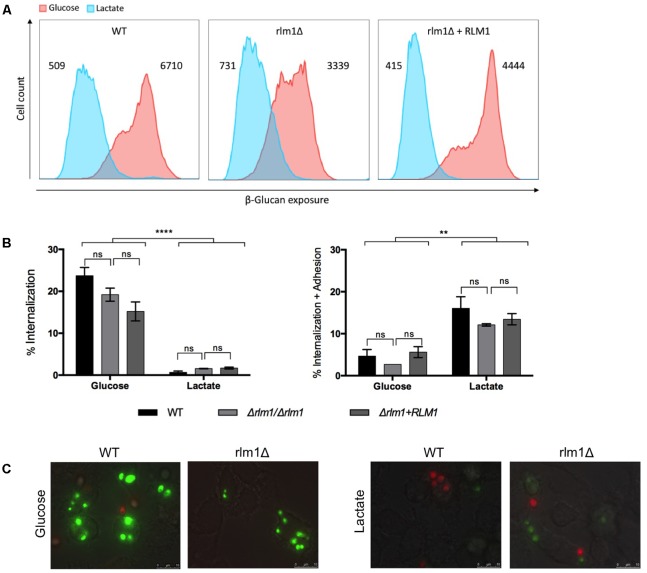
Immune recognition of *C. albicans* wild-type (WT), mutant (Δ*rlm1*/Δ*rlm1*), and complemented strains (rlm1Δ+*RLM1*). **(A)** Flow cytometry analysis of β-glucan exposure for cells grown either in YNB 2% Glucose (red) or in YNB 2% Lactate (blue). MFIs are indicated at the top of each panel and plots are representative of two independent replicate experiments (*n* = 3 per group per experiment). **(B)** Flow cytometry analysis of phagocytosis after 30 min of incubation with macrophages. Graphs represent the % of macrophages with internalized yeast cells (left), and the % of macrophages with internalized and adhered yeasts (right). ^∗^*P* < 0.05, ^∗∗^*P* < 0.01, ^∗∗∗^*P* < 0.001, and ^∗∗∗∗^*P* < 0.0001 indicate statistically significant results; ns indicates not significant. Plots are representative of three independent replicate experiments (*n* = 3 per group per experiment). **(C)** Representative micrographs showing macrophages with internalized (green labeled) or adhered (red labeled) yeast cells grown either in YNB 2% glucose or in YNB 2% Lactate.

In order to determine the importance of *C. albicans RLM1* in cell wall remodeling, we tested whether the growth of the different strains in the presence of the alternative carbon source lactate would influence the interaction with phagocytic cells (**Figure 6**). We showed previously that the absence of C. *albicans RLM1* on glucose grown cells significantly alters the proportions of the major cell wall components, enhancing the chitin content and reducing the glucans and mannans content (Delgado-Silva et al., 2014). However, nothing about the exposure of these components was previously observed. Thus, as the host immune defenses rely on the recognition of conserved molecular patterns in the fungal cell wall, particularly the glucans, we analyzed the exposure of β-glucans at the cell surface of *C. albicans* cells grown either in the presence of glucose or in the lactate. All cells were stained with anti-β-1,3-glucans and analyzed by flow cytometry. Glucose-grown cells exhibited significantly (*P* < 0.0001) higher levels of β-glucan exposure than lactate-grown cells (**Figure 6A**), consistent with previously published data (Ballou et al., 2016). Although the mutant seemed to present a different pattern of β-glucans exposure in both carbon sources, the differences were not significant.

To evaluate phagocytosis of *C. albicans* cells, we used a previously described assay (Carneiro et al., 2014), which allowed the identification of different macrophage populations by differential staining. In this way, macrophages with only internalized *C. albicans* cells (sytox green-stained) and with both internalized and surface adhered cells (PI and sytox green double stained) were clearly distinguished. As previously described, results showed that glucose-grown cells were internalized more efficiently by murine bone marrow-derived macrophages (BMDMs) compared to lactate-grown cells (**Figure 6B**) (Ene et al., 2013). In contrast, lactate-grown cells displayed higher levels of adhesion than glucose-grown cells (**Figure 6B**). However, no significant changes were observed between the wild-type and Δ*rlm1*/Δ*rlm1* mutant strains under these conditions (**Figure 6B**). Representative fluorescence microscopy analyses are shown in **Figure 6C**.

**FIGURE 7:**
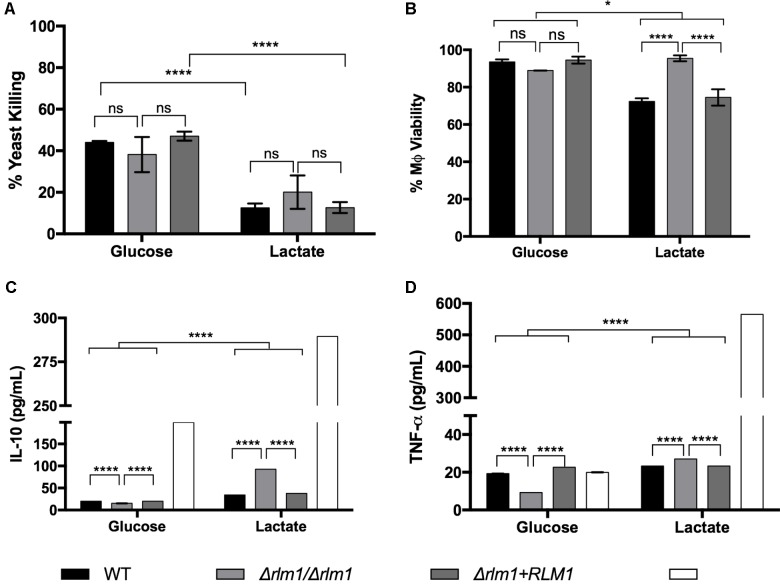
Host viability and immune response to *C. albicans* wild-type (WT), mutant (*Δrlm1/Δrlm1*), and complemented (*Δrlm1*+*RLM1*) cells grown on glucose or lactate after 1 h of infection. **(A)** Killing of yeast cells adapted to glucose or lactate and incubated with macrophages at 5:1 ratio. Results are expressed as the percentage of yeast killing. **(B)** Assessment of host viability by measuring LDH released by murine macrophages. Concentrations of **(C)** IL-10 and **(D)** TNF-α detected in culture supernatants of murine macrophages after incubation with the yeast cells. ^∗^*P* < 0.05, ^∗∗^*P* < 0.01, ^∗∗∗^*P* < 0.001, and ^∗∗∗∗^*P* < 0.0001 indicate statistically significant results; ns indicates not significant. Plots are representative of three independent replicate experiments (*n* = 3 per group per experiment). Mϕ indicates macrophages alone (near glucose) or incubated with LPS (near lactate).

Additionally, the effect of *RLM1* on phagocyte interaction and activation was assessed. For that, macrophages were infected with *C. albicans* cells, previously grown in glucose or lactate, in a MOI of 5 yeasts to 1 macrophage for 1 h. The uptake of live fungal cells by macrophages was measured by colony-forming units (CFUs) and presented in percentage of yeast killing (**Figure 7A**). Results indicated that wild-type and complemented lactate-grown cells were less efficiently killed by macrophages than their glucose counterparts (**Figure 7A**), as previously reported (Ene et al., 2013). However, no significant differences were observed between the Δ*rlm1*/Δ*rlm1* mutant strain adapted to glucose in comparison to lactate, suggesting that the mutant lost part of its resistance to killing.

The cell damage caused by the different strains was also quantified by measuring the amount of lactate dehydrogenase (LDH) released by murine macrophages after 1 h of incubation with yeast cells (**Figure 7B**). Results showed that the wild-type and complemented strains adapted to glucose led to a lower production of LDH than their lactate counterparts, resulting in higher percentage of viable macrophages (*P* < 0.001; **Figure 7B**). In contrast, the Δ*rlm1*/Δ*rlm1* mutant strain was able to cause less damage in the murine macrophages when cells where grown on lactate, in comparison with glucose, indicating that it also lost its ability to kill macrophages when grown in lactate. These results suggest that *RLM1* is involved in cell damage but only when cells are grown in lactate.

Cell activation was also evaluated by quantifying IL-10 and TNF-α after 1 h of co-incubation (**Figures 7C,D**). Results showed that glucose-grown cells stimulated less production of TNF-α (**Figure 7D**) and IL-10 (**Figure 7C**), compared to lactate-grown cells, consistent with previously published data (Ene et al., 2013). Furthermore, the Δ*rlm1*/Δ*rlm1* mutant strain grown in lactate showed higher levels of IL-10 when compared with their wild-type (*P* < 0.001) and complemented (*P* < 0.001) strains (**Figure 7C**), while when grown in glucose the results were the opposite. The same results were observed considering TNF-α secretion (**Figure 7D**), confirming that *RLM1* is relevant for immune activation. Overall, these results indicate that *RLM1* does not mediate immune recognition but is important in immune cell resistance and activation, particularly in cells grown in lactate.

**FIGURE 8:**
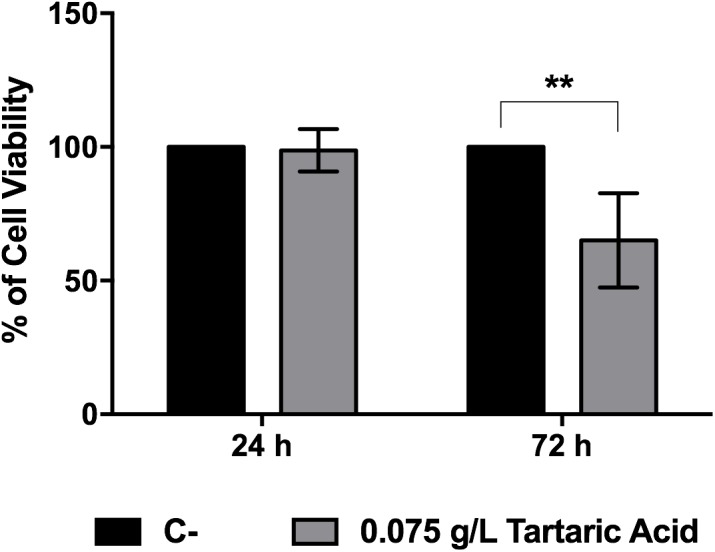
Host viability after incubation with tartaric acid. Macrophages were incubated with tartaric acid at a final concentration of 0.075 g/L for 24 and 72 h. Results express the percentage of macrophage viability. ^∗^*P* < 0.05, ^∗∗^*P* < 0.01, ^∗∗∗^*P* < 0.001, and ^∗∗∗∗^*P* < 0.0001 indicate statistically significant results; ns indicates not significant. Plots are representative of three independent replicate experiments (*n* = 3 per group per experiment). C– indicates macrophages incubated with DMEM alone.

Finally, in order to evaluate whether tartaric acid could contribute for cellular host cytotoxicity, we incubated macrophages with this organic acid and evaluate metabolic activity by MTT assay. Results showed that, after 72 h of incubation, tartaric acid reduces cellular viability by around 35% (**Figure 8**).

## Discussion

*Candida albicans* has the ability to survive and proliferate within distinct niches in the human host. This flexibility requires a rapid adaptation to local conditions, forcing the pathogen to utilize the alternative nutrients that are available. Some of these niches contain glucose, the preferred carbon source of *C. albicans*, while others contain different carboxylic acids such as lactate (Owen and Katz, 1999; Barelle et al., 2006). These fluctuations on carbon sources affect profoundly the physiology of *C. albicans*, including alterations in the cell wall structure that impact immune recognition (Ene et al., 2012a, 2013; Ballou et al., 2016). The transcription factor *RLM1* has been shown to play an important role in the maintenance of the cell wall, affecting the adhesion ability and virulence (Bruno et al., 2006; Delgado-Silva et al., 2014). However, the *in vitro* dissection of these observations has been performed using *C. albicans* cells grown on artificial growth media containing 2% glucose, a condition that does not accurately reflect the host niches where this fungus can proliferate. Therefore, we studied the role of *C. albicans RLM1* during growth on the physiologically relevant nutrient lactate, using a set of previously constructed Δ*rlm1*/Δ*rlm1* mutants (Delgado-Silva et al., 2014).

First, we characterized the growth and metabolism of all strains grown in glucose or in lactate (**Figures 1**, **2**). As expected, the growth rate of cells grown in glucose was much higher than cell grown in lactate. The absence of a functional *RLM1* did not affect growth rate in both carbon sources. We observed that cells grown in glucose were able to consume the totality of this carbon source within the first 20 h of growth, and produced glycerol, ethanol, and acetic acid, all known fermentative metabolites. However, when cells were grown in the presence of lactic acid, they consume only a small part of the carbon source and none of the above metabolites were observed, but tartaric acid was detected (**Figure 2**). Tartaric acid is an analog of malic acid, which is a key intermediate in the cycle of Krebs. The production of tartaric acid when yeast cells were grown in the presence of lactate may be a way of the yeast to regulate carbon flow through the cycle of Krebs. Moreover, since tartaric acid was excreted, this may affect the cycle of Krebs of the host cells, reducing the energy aerobically produced by oxidizing glucose. Instead, the host cells may convert glucose into lactic acid anaerobically, in a positive feedback loop. This overproduction of lactate has a direct toxic effect on muscles, which may explain some symptoms associated with *Candida* overgrow, such as fibromyalgia (Garrison and Breeding, 2003). Nevertheless, these observations were controversial as no evidence has been found for the production of tartaric acid as a metabolic end product by *Candida* (Lord et al., 2005). Here, we show that *C. albicans* produces tartaric acid when growing in the presence of lactate and this metabolite, at a concentration identified in our studies, reduces cell viability. This is particularly interesting as lactate is present in several niches within the host, including within the gastrointestinal tract and vagina (Owen and Katz, 1999; Barelle et al., 2006), suggesting that when cells grow in these niches, the production of tartaric acid may explain some symptoms associated with *Candida* infections.

We then compared the same typical cell wall phenotypes described for glucose-grown *C. albicans* Δ*rlm1*/Δ*rlm1* mutant cells by performing the tests in parallel with the control strains grown in the presence of lactate (**Figure 3**). We found that lactate-grown cells, independently of the mutation, presented hypersensitivity to different stresses that affected the cell wall, such as Caffeine, Caspofungin, and SDS (**Figure 3B**). The cell wall of *C. albicans* lactate-grown cells is described as being thinner, presenting less β-glucans and mannans and is more porous than their glucose counterparts (Ene et al., 2012a,b). The differences observed between the two conditions reflect the alterations in the cell wall composition due to the carbon source and the porosity may explain the complete absence of growth of lactate-adapted cells on SDS. Since these phenotypes were similar for all strains, it is probable that similar cell wall alterations observed previously (Ene et al., 2012a,b) may also occur in the mutant strain. Moreover, in lactate grown cells, the absence of a functional *RLM1* reverted the hypersensitivity to Congo Red (**Figure 3B**) observed when cells were grown in glucose (**Figure 3A**). It has been described that Congo Red interacts with cell wall polysaccharides, exhibiting a particularly high affinity for chitin and β-glucans (Klis et al., 2002). Thus, the fact that in lactate-grown cells, β-glucans are masked may reduce the availability of Congo Red to β-glucans, rendering the mutant more resistant.

*Candida albicans* has evolved multiple strategies, including the expression of several virulence factors, to overcome the different environmental conditions imposed by the host (Calderone and Fonzi, 2001; Mayer et al., 2013). As many of these factors rely on morphology changes, we also tested whether *RLM1* could be involved in some virulence mechanisms such as hyphal growth, adhesion and biofilm formation during carbon adaptation. Under our growth conditions, we showed that the Δ*rlm1*/Δ*rlm1* strain presented shorter hypha than the WT or complemented strains when grown in glucose as well as in lactose, indicating that *RLM1* is involved in *C. albicans* filamentation independently of the carbon source (**Figure 4**). As we reported previously, the Δ*rlm1*/Δ*rlm1* strain showed a reduction in the cell wall mannans (Delgado-Silva et al., 2014) and loss of cell wall O-mannans was associated with impaired hyphal growth (McKenzie et al., 2010). Thus, the defect in filamentation observed could be correlated with the cell wall composition.

Additionally, *C. albicans RLM1* has been described as a negative regulator of *in vitro* biofilm formation, as the Δ*rlm1*/Δ*rlm1* mutant strain forms more biofilm than the wild-type in presence of glucose (Delgado-Silva et al., 2014). This gene also seems to regulate negatively some of the same targets of *BCR1*, a well-known transcription factor that governs biofilm formation (Nobile et al., 2006). We observed that in the presence of lactate, all strains produced more biofilm, but as previously reported, the Δ*rlm1*/Δ*rlm1* strain formed even more biofilm, than in the presence of glucose (**Figure 5**). This enhanced biofilm formation in lactate may be directly correlated with the higher ability (around three times more) of the lactate grown cells to adhere. These results indicate that the role of *RLM1* as a negative regulator of *in vitro* biofilm formation is independent of the carbon source.

Finally, we studied the involvement of *RLM1* in host-pathogen recognition. The cell wall of microbial pathogens is the first point of contact with the host defenses. Then, any modification on the cell surface, especially on the pathogen-associated molecular patterns (PAMPs), such as β-glucans, α-, and β-mannans, phosphomannans and chitin, impacts the immune detection. The host metabolite lactate has been shown to modulate the exposure of some PAMPs in *C. albicans*, affecting the recognition of the fungus by the host phagocytes (Ene et al., 2012a, 2013; Brown et al., 2014; Ballou et al., 2016). During growth in lactate, the β-glucans are actively masked by the outer mannans layer (Ballou et al., 2016).

In our experiments, *RLM1* does not seem to be involved in β-glucans masking (**Figure 6A**), which is consistent with the phagocytosis results, since no significant differences were observed between the wild-type and the Δ*rlm1*/Δ*rlm1* mutant in both carbon sources (**Figures 6B,C**). Moreover, our results confirmed that cells grown in lactate were less internalized when compared with their glucose counterparts (**Figure 6**) (Ballou et al., 2016), but remained attached to the macrophages (**Figure 6B**). Once more *RLM1* does not seem to be involved in this mechanism.

Although no significant differences were observed in the percentage of yeast killing between Δ*rlm1*/Δ*rlm1* and their counterpart strains, within each condition (carbon source), our results confirm previous data that lactate grown cells are more resistance to phagocyte killing (Ene et al., 2013). However, it is curious to observe that when the mutant was grown in lactate, the resistance to yeast killing was lower and not statistically different when grown in glucose, indicating that without *RLM1 C. albicans* loses its resistance acquired when cells grow in lactate (**Figure 7A**). Since previous results indicate that *RLM1* is involved in cell wall architecture, when cells were grown in glucose, this loss of resistance to killing could also be due to changes in cell wall structure and composition and account for its lower virulence in the disseminated mouse model of infection (Delgado-Silva et al., 2014). The levels of LDH released by macrophages, an indicator of cell damage, were increased for the cells grown in the presence of lactate, indicating that these cells although being taking up by the macrophages less efficiently, are more prone to kill these phagocytic cells (**Figure 7B**). Interestingly, this is not verified for the Δ*rlm1*/Δ*rlm1* mutant. As seen previously (**Figure 7A**), the mutant is more susceptible to macrophage killing, which explains the lower ability to escape from macrophages by damaging the phagocyte. Previous data demonstrated that hyphal extension is a key factor promoting fungal escape from phagocytes, therefore the fact that Δ*rlm1*/Δ*rlm1* mutant produces shorter hyphae might also contribute to the lower capacity to damage macrophages.

The amount of IL-10 and TNF-α was also quantified in order to evaluate the ability of the fungus to induce anti- and pro-inflammatory responses, respectively (**Figures 7C,D**). As expected, the cytokines profile of the *C. albicans* in lactate grown cells points to an anti-inflammatory response, given the increased levels of IL-10 in this conditions. However, the levels of IL-10 in the Δ*rlm1*/Δ*rlm1* mutant were even higher. The masking of β-glucans on the surface of lactate grown cells reduces not only the phagocytosis, which is stimulated via dectin-1, but also the secretion of cytokines, which is under the control of the transduction pathways upon activation of dectin-1 (Steele et al., 2003). In our study, we observed that the levels of TNF-α were lower with the mutant, when compared with the wild-type and complemented strains, in glucose-grown cells, but slightly higher in lactate-grown cells. This would suggest that β-glucans in the mutant strain grown in glucose would be less exposed than their counterparts while in lactate-grown cells it would be the opposite. Regarding IL-10, the higher concentration observed when macrophages were incubated with the mutant cells grown in lactate, could also be explained by the fact that the β-glucans’ masking of the mutant was much lower than in the wild-type and complemented strains. However, and once more, the differences were not significant. The fact that interpreting relative MFI for individual runs across strains is sensitive to growth rate, growth phase, staining uptake, and cell size distribution could contribute to not reach statistical significance. These slight differences in β-glucans exposure would not influence phagocytosis but could account for the differences in host cell activation.

Taken together, these results confirmed that *C. albicans* cells grown in the presence of lactate were less internalized and killed by macrophages and suggest that *RLM1*, although not being involved in yeast cells internalization, seems to be involved in the killing by macrophages and inflicting damage to host cells, what could be related to the lower capacity of the mutant to filament. This interaction mediates cytokine levels, rendering the lactate-grown cells less visible to the phagocytic cells, as previously reported (Ene et al., 2013). However, although the Δ*rlm1*/Δ*rlm1* induced a higher anti-inflammatory response, the modifications in the cell wall rendered the mutant less resistant to action of the immune system.

## Conclusion

This study reveals that regardless of the carbon source, *C. albicans RLM1* is involved in filamentation and biofilm formation, which could be directly correlated with the architecture and composition of the cell wall. It also showed that some phenotypes were dependent on the carbon source, such as resistance to macrophage killing and ability to damage macrophages, which can also be correlated to changes in the cell wall. Thus, the carbon source that presents in different host niches, such as lactate, affects the remodeling of the *C. albicans* cell wall, with the potential to also modulate host metabolism, and suggests that *RLM1* plays an important role in this pathway.

## Author Contributions

PS and CP designed the experiments. JO-P, RA, BC-R, AC-B, and PP-S carried out the experiments. JO-P, RA, BC-R, CP, and PS analyzed and interpreted the data. JO-P, RA, and PS wrote the manuscript with additional input from BC-R, SP, SS, MH, and CP.

## Conflict of Interest Statement

The authors declare that the research was conducted in the absence of any commercial or financial relationships that could be construed as a potential conflict of interest.
